# Method Matters: Enhancing Voice-Based Depression Detection With a New Data Collection Framework

**DOI:** 10.1155/da/4839334

**Published:** 2025-05-20

**Authors:** Dan Vilenchik, Julie Cwikel, Yacov Ezra, Tuvia Hausdorff, Mor Lazarov, Ruslan Sergienko, Rachel Abramovitz, Ilana Schmidt, Alison Stern Perez

**Affiliations:** ^1^School of Electrical and Computer Engineering, Ben Gurion University of the Negev, POB 653, Beer Sheva 84105, Israel; ^2^Center for Women's Health Studies and Promotion, Ben Gurion University of the Negev, POB 653, Beer Sheva 84105, Israel; ^3^Functional Neurology Out-patient Clinic, Soroka University Medical Center, Beer Sheva, Israel; ^4^Department of Computer Science, Ben Gurion University of the Negev, POB 653, Beer Sheva 84105, Israel; ^5^Department of Health Policy and Management, Faculty of Health Sciences, Ben-Gurion University of the Negev, POB 653, Beer Sheva 84105, Israel

## Abstract

Depression accounts for a major share of global disability-adjusted life-years (DALYs). Diagnosis typically requires a psychiatrist or lengthy self-assessments, which can be challenging for symptomatic individuals. Developing reliable, noninvasive, and accessible detection methods is a healthcare priority. Voice analysis offers a promising approach for early depression detection, potentially improving treatment access and reducing costs. This paper presents a novel pipeline for depression detection that addresses several critical challenges in the field, including data imbalance, label quality, and model generalizability. Our study utilizes a high-quality, high-depression-prevalence dataset collected from a specialized chronic pain clinic, enabling robust depression detection even with a limited sample size. We obtained a lift in the accuracy of up to 15% over the 50–50 baseline in our 52-patient dataset using a 3-fold cross-validation test (which means the train set is *n* = 34, std 2.8%, *p*-value 0.01). We further show that combining voice-only acoustic features with a single self-report question (subject unit of distress [SUDs]) significantly improves predictive accuracy. While relying on SUDs is not always good practice, our data collection setting lacked incentives to misrepresent depression status; SUDs were highly reliable, giving 86% accuracy; adding acoustic features raises it to 92%, exceeding the stand-alone potential of SUDs with a *p*-value 0.1. Further data collection will enhance accuracy, supporting a rapid, noninvasive depression detection method that overcomes clinical barriers. These findings offer a promising tool for early depression detection across clinical settings.

## 1. Introduction

According to recent analyses, 16% of the global disability-adjusted life years lost are attributable to mental disorders, especially depression. The economic loss associated with this burden of disease is estimated at 5 trillion USD per year [1]. Yet, the majority of those persons are neither recognized, diagnosed, nor adequately treated [2–4], prompting a WHO report to call for improved mental health detection, especially for depression and anxiety [5]. Most persons who do seek attention for symptoms of depression or anxiety will do so within the primary health care system rather than seek specialized psychiatric care, which is harder to access and more expensive [6–9].

However, the accurate detection of mental health problems in the community and their treatment remains a challenging and costly situation, burdening the mental health care systems, which often lack the manpower, training, and other resources to provide effective treatment [2–4, 10]. In general, the diagnosis of mental health conditions, such as depression and anxiety, requires the use of validated questionnaires or a personal interview by a mental health professional, usually a psychiatrist [2, 11]. Therefore, developing an easily used method for detecting depression should provide a way for early and accurate diagnosis, leading to better treatment overall, with lower costs and higher rates of detection, treatment, and recovery [6, 12–20].

Voice analysis is an attractive diagnostic tool, offering clinicians a noninvasive, objective, and scalable method for early detection and continuous monitoring of depression, which can enable timely interventions and improved mental health outcomes. In modern practice, voice analysis is typically conducted through machine learning (ML) pipelines trained to predict specific conditions using voice as a primary input modality, sometimes alongside other data modalities such as text, sensor data, or self-report measures. These pipelines leverage the changes in acoustic characteristics associated with depressive symptoms, positioning voice as a valuable biomarker for depression triage and as a tool for diagnostic decision-making.

Research groups in academia and industry are working on vocal biomarkers for physical conditions from Parkinson's [21] to coronary heart disease [22] and, more recently, COVID-19 [23]. In the area of mental health, there are studies utilizing voice to detect PTSD [24], suicide risk [25, 26], psychosis [27, 28], bipolar disorder [29], and depression [19, 30–40]. As this suggests, there has been extensive research on depression detection using voice, making it impractical to individually describe all cited recent works. We refer the reader to a comprehensive review of using acoustic features for depression detection [41].

However, reviewing the ML methods used in recent studies (e.g., [34, 37–40]), we can highlight that our present study stands out due to its unique combination of (1) the use of short voice samples (1 min), (2) high-accuracy label acquisition through personal interviews conducted by social workers with mental health training, (3) the integration of subject unit of distress (SUDs) scores as an input feature, demonstrating how voice data enhances predictions beyond SUDs alone, highlighting its realistic application in clinical scenarios requiring high accuracy, and (4) a focus on a specialized population with chronic pain. This population offers a unique combination of high depression prevalence (~50%) and generalizability, as chronic pain affects a broad cross-section of the population [42]. Importantly, studies suggest that chronic pain is likely not to confound voice analysis, as individuals tend to adapt to the pain over time, ensuring that the voice features primarily reflect depression rather than pain [43, 44].

Elaborating on item (4), let us note that the majority of papers report how many depressed persons were recruited for their study, but they fail to report how many were interviewed to get to this number of depressed persons, for example [39, 40] (in the worst case, about 20 times more, as this is the prevalence of depression in the general population—5%). In this paper, we report the total number of persons included in the study sample, depressed or not. This emphasizes our method's efficient nature, highlighting its applicability in large-scale data collection.

## 2. Methods

In this study, we concentrated on addressing some of the salient problems in voice analysis for mental health symptom detection, especially in the case of depression. These are described in this section to highlight what we did differently in the present study. Existing studies suffer from several inherent challenges, which are often ignored in most papers; some are unique to voice data, but many are relevant to all ML studies in mental health.

A. Data imbalance. The key challenge in collecting data from depressed people is the a priori low prevalence of depression in the population. Depression affects ~5% of adults globally, with a higher prevalence among women (6%) than men (4%), according to the WHO [45]. In the U.S., the NIMH reports that 8.3% of adults experienced a major depressive episode in the past year, with rates higher among young adults aged 18–25 (18.6%) and females (10.3%) compared to males (6.2%) [45]. On top of that, depressed individuals often experience dysphoric mood, low motivation, and psychomotor issues, making them less likely to leave home or participate in surveys [39, 46–49]. This limits the availability of depressed voice samples in real-world settings, often resulting in highly imbalanced datasets (e.g., 80–20 class ratios). Such datasets pose significant ML training challenges, affecting accuracy, precision, and recall. Despite deep learning and big data advancements, a recent survey confirms the issue remains unsolved [50].
B. The quality of the label. Depression classification requires labeled data, obtained through self-reports or clinically validated questionnaires like PHQ-8 [51] and CES-D [52]. While validated questionnaires are essential for clinical applications, they limit participant pools compared to simpler measures like SUDs [53]. Furthermore, data collection without direct contact with respondents, as in remote online surveys, poses reliability concerns, as respondents can submit multiple or false entries. Similarly, bot-administered interviews lack behavioral observation, raising doubts about data validity. Symptom misreporting is well-documented—for example, new mothers, especially from minority or refugee backgrounds, often underreport postpartum depression due to fear of losing custody of their infants [54, 55].
C. Homogeneity of the sample. A possible solution to issues A + B is recruiting respondents from controlled groups, such as students, veterans with PTSD, or elderly residents in care facilities [24, 25, 40, 47]. While this addresses data availability and reliability, it creates homogeneous samples, limiting model generalizability and reducing practical applicability. Homogeneity also arises from single-gender or single-ethnicity datasets [24, 45, 49]. Some studies suggest separate models for different demographics [26, 47], while others advocate for cross-cultural methods [56, 57]. For clinical use (e.g., by family doctors in HMOs), broader generalization is crucial for real-world adoption.
D. Hardware. Different recording devices (microphones, smartphones) use varying hardware and compression algorithms, altering acoustic properties and introducing device-specific distortions. These inconsistencies can reduce classification model performance, making it harder to generalize across devices and impacting tasks like emotion detection and speech recognition [58]. The issue is especially problematic in remote data collection, where participants use their own phones or webcams, and recordings may undergo compression (e.g., via Zoom, WhatsApp, or cloud uploads). Standardizing recording conditions or adopting device-agnostic processing is crucial for robust voice-based ML applications.

To demonstrate that these challenges affect real-world voice studies on depression detection, we examine the DAIC-WOZ dataset, which is widely used in automatic depression detection. It consists of 189 clinical interviews conducted by a virtual Bot, Ellie, which may improve disclosure in some areas but is less effective at eliciting mood and anxiety symptoms [59, 60]. The dataset is imbalanced (107 nondepressed, 35 depressed) and split into 70–30 stratified train-test (Concern A). Maio et al. [61] found that voice-only models achieved 85% accuracy, just 10% above a naïve classifier (75%). Depression labels relied on PHQ-9 [62, 63], but administration details are unclear, raising concerns about label reliability (Concern B). Generalizability (Concern C) is also an issue, as participants were mostly U.S. veterans from Los Angeles [64]. Finally, hardware variability is undocumented, making it unclear whether consistent recording setups were used (Concern D). This likely explains why no studies have applied DAIC-WOZ to external data—researchers either train and test on DAIC-WOZ or ignore it entirely. Furthermore, no clinical applications have been developed from the DAIC-WOZ data set.

To the best of our knowledge, no group has surmounted the technical challenges of moving from small studies to large-scale integrated clinical applications for mental health detection. The present paper offers an end-to-end data collection and analysis pipeline that methodologically deals with the abovementioned four issues.

## 3. Data Collection

We aimed to bridge these methodological gaps by detecting depression within the general population using a small but high-quality dataset with a very high prevalence of depression.

Our data was collected from an outpatient chronic pain clinic adjacent to Soroka University Medical Center, where there is a high percentage of persons with symptoms of either depression or anxiety, yet clinic attendees are not considered psychiatric patients. This is significant given that we are developing an algorithm to detect these conditions before a psychiatrist sees the patient. The setting was chosen to address the challenge of gathering a substantial number of depressed individuals in a small sample, given the high prevalence of depression among chronic pain patients [65]. While we collected data on anxiety symptoms as well as depression, in the present paper, we present the depression findings only.

We collaborated with a chronic pain specialist (third author) who maintains a regular weekly functional neurology clinic (this is the clinic's name). One may argue that this approach targets a specific population rather than a truly general one. However, two critical points justify our methodology. First, chronic pain afflicts all walks of society, regardless of race, ethnicity, gender, or socioeconomic status. In addition, the clinic belongs to the largest of the four HMOs in Israel, servicing over 2 million patients and providing affordable health services to 60% of the population of Israel. Thus, we could reach a wide and heterogeneous pool of patients while conditioning on a second label, pain. This answers Concern (B) (data homogeneity).

Second, one will probably throw a wrench into the works at this point, objecting that we add a confounder, pain, which interferes with the depression/anxiety signal. In depression detection using voice samples, a critical consideration is the potential confounding effect of pain on acoustic signals. However, our study, which focuses on outpatients in a chronic pain clinic, found that the pain acoustic signal did not interfere with the detection of depression in this ambulatory population.

This finding is supported by the notion that chronic pain and its manifestation in voice may differ significantly from acute or momentary pain. Prior research indicates that pain can indeed be detected in voice; for instance, studies by Oshrat et al. and Borna et al. [66, 67] demonstrated the feasibility of identifying pain through vocal characteristics. However, these works primarily examined subjects experiencing acute pain, which may present distinct vocal markers compared to chronic pain [68].

Chronic pain is characterized by a persistent, long-term experience, which may lead to an adaptation in vocal expression, potentially diminishing its impact on acoustic features used for depression detection [43, 44]. Our findings suggest that the vocal adaptation associated with chronic pain allows for the isolation of depression-related acoustic features without significant interference from pain-related signals.

Interviews were conducted in person by trained interviewers with a background in mental health, and the respondents signed informed consent forms in the presence of the collaborating physician. Interviewers were supervised on how to encourage the response rate, which was 62% of those approached. Of those who gave an initial agreement and signed informed consent forms, 52% completed the full interview. This is comparable with known response rates for studies recruiting from clinical settings (e.g., hospitals and clinics), which report response rates typically ranging from 40% to 70% [69]. This higher response rate can be attributed to the accessibility of potential participants, the interpersonal sensitivity of the interviewers, and the involvement of healthcare providers in the recruitment process. For comparison, when recruiting from the general community, response rates tend to be lower, often ranging from 20% to 50%. Online recruitment rates are on the lower end, around 10%–30%, depending on the outreach strategy and the incentives provided [70].

In our procedure, the patient was asked to give a personal narrative to answer factual questions (e.g., how is the weather?, how they arrived at the clinic?, What clothes they are wearing?, etc.), and their voice was recorded. The interviews were conducted in a quiet room with a consistent recording device (iPad, using the Voice Record Pro software).

The presence of depression was determined by a commonly used depression questionnaire (CES-D Depression Scale—short form) [52] with its validated clinical cut-off [71]. Again, we chose this questionnaire for its brevity, clinical validity, and because it does not require a trained clinician to administer.

Pain levels were measured using the PHQ-15 (Patient Health Questionnaire -15)—aka the Somatic Symptom Severity Scale, which is a self-report version of the Primary Care Evaluation of Mental Disorders (PRIME-MD) [62] with a validated cut-off, which is a different instrument than the PHQ-8 (mentioned above as an oft-used measure of depression, e.g., [63]).

### 3.1. Adding SUDs and Pain Scores

In addition to the two clinically validated mental health questionnaires (CES-D for depression and PHQ-15 for pain), we included subjective measures of depression using one single question based on the SUDs format [53, 72], with 1 being no depression and 10 being very high levels. The SUDs were not used to determine the label; only the clinically validated questionnaire was used for the label. This meets Concern (C), label validity.

Ethical considerations: Participants received an explanation of the format of the study and provided written informed consent. The study was approved by the Spitzer Department of Social Work ethics committee at Ben Gurion University of the Negev and by the participating hospital Helsinki committee (Soroka University Medical Center).

## 4. Data Description

Our dataset contains samples from 143 patients, 123 women (86%) and 20 men (14%). The average age was 52.3 years (SD = 14.6), 61% were born in Israel, and 39% immigrated from other countries. The average years of education were 14 years (SD = 0.26). Most of the sample (61%) were married or living with a partner.

Of the total sample, 62.5% had depression based on the clinical research instrument (CES-D) detailed above. In this study, we focus primarily on the women, for reasons that will become clear during the analysis. Out of the 123 women in the study, 64% have depression. Based on the PHQ15 results from the total sample, 12.7% had minimal or mild pain symptoms, 19.7% had moderate pain, and 67.5% had severe pain symptoms.

**Figure 1:**
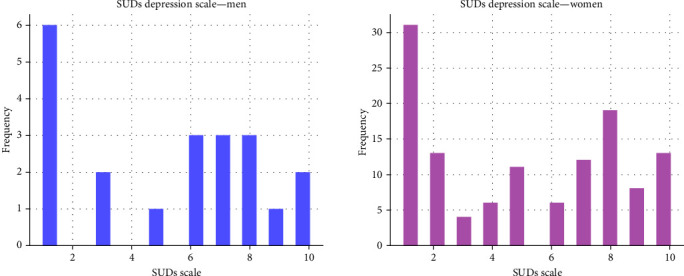
SUDs depression scale distribution for men and women, the mean is around 5 for both groups.

Figure 1 shows the SUDs depression score distribution for the *men* and *women* samples. The women's group with low SUDs (score of 1) and high SUDs (score of 9,10) we call this group the Low-High SUDs group, or LH for short. The LH-depression group included 52 patients, and it represents 42.3% of all women. In this group, exactly 50% of the women are depressed.

The correlation between depression SUD scores and PHQ-15 is 0.5, with a *p*-value of 0.005. The correlation in the restricted LH group is 0.48. Note that this correlation is between the labels and not between the acoustic features. The correlation between the SUDs scores and CES-D is 0.725. For the women's group, it's 0.746, and for the men, 0.635. In LH-Group, the correlation is 0.778.

In this paper, we analyze two groups: the entire women's dataset and the LH-Group (Low-High SUDS).  Table 1 gives the details of the two groups.

## 5. Preprocessing and Feature Extraction

**Figure 2:**
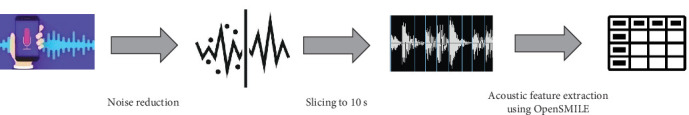
The feature processing and extraction pipeline, taking a raw voice recording and processing it into a table of acoustic features.

The preprocessing step included three main components: Noise reduction, slicing, and acoustic feature extraction using OpenSMILE (Open Speech and Music Interpretation by Large-space Extraction; Figure 2).

All the recordings were filtered from noise using the logMMSE noise reduction algorithm, implemented in Python's logMMSE package. The logMMSE noise reduction library in Python is designed to enhance the quality of audio recordings by mitigating the impact of background noise. This library employs the logMMSE (logarithmic minimum mean square error) algorithm, a sophisticated noise suppression technique. The algorithm transforms the noisy audio signal into the log-spectral domain, estimates the clean speech signal, and then inversely transforms it back to the time domain. This process effectively reduces noise while preserving the integrity of the speech signal. We accessed the logMMSE Python package through its simple API.

After filtering noise, we segmented each recording into standardized chunks of 10 s each. To ensure that the resulting data is informative and focused on relevant speech components, we employ an in-house trained support vector machine (SVM) model to detect and remove segments of silence from these chunks. Although the resulting chunks from the same person are correlated, this process results in a dataset about five times larger, with ~5–6 chunks per patient (see Table 1). Despite the correlation, this augmentation provides more freedom in classifying the test data and increases the amount of data available for training, ultimately improving the accuracy of our model. In the next section, we'll describe how this fact was used to classify the test samples.

## 6. Feature Extraction and Features Description

Low-level descriptor (LLD) features were extracted from each chunk using the OpenSMILE open-source toolkit. LLDs refer to basic acoustic features extracted from audio signals. These features capture fundamental audio signal properties that can be used to analyze and understand various aspects of the speaker's voice, such as emotional state, stress level, and other affective characteristics. LLDs are typically calculated over short audio signal frames and provide detailed information about the signal's behavior over time. Some common types of LLDs include:

• Pitch (F0): The fundamental frequency of the voice, which correlates with perceived pitch.
• Energy: The loudness or intensity of the audio signal.
• Formants: Resonant frequencies of the vocal tract, which contribute to the timbre of the voice.
• Mel-frequency cepstral coefficients (MFCCs): Representations of the short-term power spectrum of the audio signal.
• Harmonics-to-noise ratio (HNR): The ratio of harmonics to noise in the voice, indicating voice quality.
• Jitter and Shimmer: Measures of frequency and amplitude variations in the voice, respectively.

OpenSMILE is a versatile and widely used open-source toolkit designed to extract features from audio signals [73]. Developed by the Technical University of Munich, OpenSMILE is renowned for its capability to analyze speech and music, providing an extensive array of LLDs and functionals that characterize various properties of audio recordings. OpenSMILE offers a range of pre-configured feature sets, such as Extended Geneva Minimalistic Acoustic Parameter Set (eGeMAPS) and the large OpenSMILE emotion feature set, which are tailored for specific tasks like emotion recognition, stress detection, and health monitoring. We used the following feature sets to extract the LLDs:

1. eGeMAPS: A minimalistic feature set containing 88 features, selected for their potential to index affective physiological changes in voice production, their proven value in former studies, their automatic extractability, and their theoretical significance.
2. The large OpenSMILE emotion feature set: A comprehensive feature set with 6552 features enabled.

## 7. The Experimental Setting

### 7.1. Training the ML Algorithm

In this study, we used the CatBoost algorithm [74]. CatBoost is a gradient boosting algorithm that implements Ordered Boosting to mitigate overfitting, making it particularly effective on small datasets. We tried various Random Forest and Boosted models, and CatBoost performed the best overall; hence, we are only reporting these results. The hyperparameters for the CatBoost model were set as follows: Iterations: 100, Learning Rate: 0.05, Depth: 3, Auto Class Weights: Balanced.

**Figure 3:**
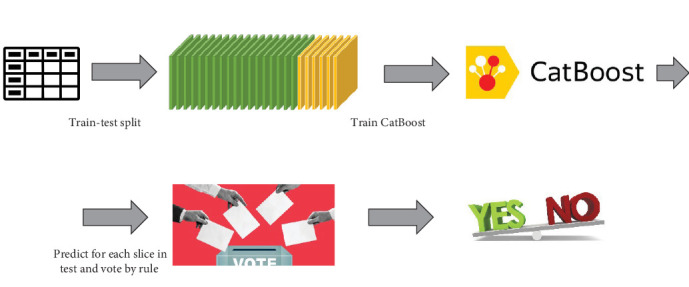
The train-test pipeline using the preprocessed data. Acoustic features used to train an ML classifier (CatBoost); At test time, CatBoost is applied to all snippets of a patient voice recording, and a threshold is applied to the aggregated “votes” to give a binary decision.

Given a training set *D* of patients' voice recordings, the recordings were processed using the pipeline in Figure 3. As a result, each patient's voice recording was broken into chunks, each processed separately using OpenSMILE. The patients were split into train and test sets, where all chunks of the train patients were gathered in one *m* × *f* table, *T*, where *m* the total number of chunks and *f* the total number of features (depending on which feature set was chosen for OpenSMILE). Similarly, table *T*' was created for test patients. Finally, we added the binary label column to Table *T*, with depression or without.

While our primary focus is on voice-only analysis, we included SUDs as a complementary reference measure in a subset of experiments. In our clinical setting, patients had already scheduled appointments and were about to see their treating physician, minimizing any incentive to misreport their emotional state. Although not central to our approach, including SUDs helped contextualize the voice-based predictions in a setting where self-report bias was unlikely.

Given that SUDs and CES-D scores correlate highly (*r* = 0.78) yet are not identical, it is a reasonable research question, never studied before in the literature, to examine how much acoustic features contribute beyond SUDs.

### 7.2. Testing the ML Algorithm

After the CatBoost model was trained, we used it to predict the label on the test set. Recall that test set patients also had several chunks per person. So, for each patient *i* we have a vector *c*_*i*_ = (*c*_*i*1_, *c*_*i*2_,…, *c*_*ir*_) of predictions, one prediction for each chunk *j*; *c*_*ij*_ = 1 if the chunk tested positive for the target label (say, positive for depression) and 0 otherwise. Now, a single prediction needs to be made. We chose to use a threshold for the decision. Namely, given a threshold *τ* (the same threshold for all patients), we predict patient *i* to be positive for the target label if at least *τ* of their chunks were. Formally, for patient *i*, label *l*_*i*_ = 1 if $\sum_{j=1}^{r}c_{ij}\geq \tau$.

The threshold values we checked were one to four. The value of the threshold governs the specificity-sensitivity tradeoff. The larger *τ* is, the more evidence we require before classifying a patient as depressed. This reduces sensitivity and increases specificity. A smaller threshold has the opposite effect.

## 8. Results

The results presented in this section were obtained via a k-fold cross-validation test. k-fold cross-validation (k-fold CV) is a resampling technique used to evaluate the performance of a ML model. The dataset is divided into equally sized folds (subsets) of *k*. The model is trained *k* times, using (k−1) folds for training and the remaining one-fold for testing. The results from each fold are averaged to provide an overall performance metric, which assesses the model's ability to generalize to unseen data. Given the small size of our dataset, the choice of k in cross-validation can significantly impact performance. A larger *k* (e.g., *k* = 4) results in smaller test sets, which may yield less stable and informative performance estimates. Conversely, a smaller k (e.g., *k* = 2) reduces the training set size to only 50%, hindering learning and leading to suboptimal results. To assess the robustness of our findings across different training and test set sizes, we report *k* = 2, 3, and 4 results.

For completeness, we briefly define each metric. The reader may skip this part if this information is redundant.

Accuracy: The ratio of correctly predicted instances, true positives (TP), and true negatives (TN) to the total number of samples. It measures the overall correctness of the model.

  $$\begin{array}{c} \text{Accuracy}=\left(\text{TP}+\text{TN}\right)/\text{NumSamples}. \end{array}$$

Precision: The ratio of TP predictions to the total number of positive predictions (TP and false positives [FP]). It measures the accuracy of positive predictions.

  $$\begin{array}{c} \text{Precision}=\text{TP}/\left(\text{TP}+\text{FP}\right). \end{array}$$

Sensitivity (Recall or TP Rate): The ratio of TP predictions to the total number of actual positives (TP and false negatives [FN]). It measures the ability of the model to identify positive instances.

  $$\begin{array}{c} \text{Sensitivity}=\text{TP}/\left(\text{TP}+\text{FN}\right). \end{array}$$

Specificity (TN Rate): The ratio of TN predictions to the total number of actual negatives (TN and FP). It measures the ability of the model to identify negative instances.

  $$\begin{array}{c} \text{Specificity}=\text{TN}/\left(\text{TN}+\text{FP}\right). \end{array}$$

Alongside the model's performance parameters, we also report:

• Baseline Accuracy: The accuracy of a simple model that always predicts the majority class or uses the prior distribution without learning from the features. This serves as the minimum benchmark for model performance.
• Accuracy Lift: The difference between the model's and baseline accuracy indicates the additional predictive power gained by using the trained model instead of the baseline guess.

## 9. Results for All Data (Men + Women)

**Figure 4:**
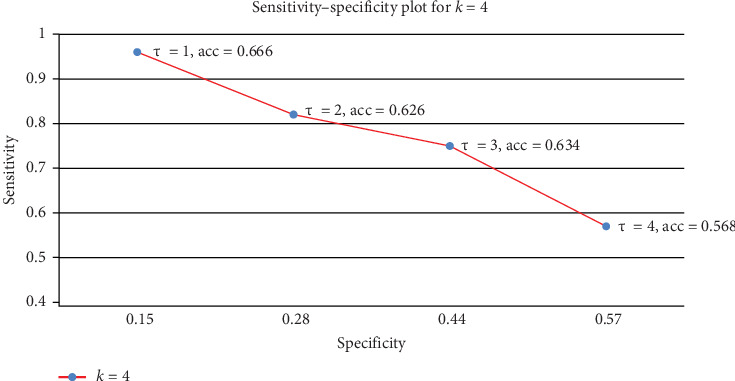
The sensitivity–specificity tradeoff for the entire data, using a 4-CV test, as the threshold *τ* varies from 1 to 4. Best accuracy is obtained at *τ* = 1. The baseline accuracy is 64.2%; the best accuracy we obtained is 66.6%, a 2% lift. The standard deviation across folds is 2%–3% depending on *τ*.

Figure 4 summarizes the results for the entire data. The figure was generated by extracting key performance metrics, sensitivity, specificity, and accuracy from the raw data in Table A1. Table A1 summarizes the results using the eGeMAPS feature set. Tables A1 and A2 (Table A2 is the same as Table A1, just with a different feature set, EmoLarge) are in the appendix. The most stable results are observed for *k* = 4, where 75% of the data is used for training. This larger training set reduces variance and provides more consistent outcomes, as detailed in the accompanying tables. Therefore, Figure 4 plots only the data for *k* = 4. The remaining *k* = 2,3 values can be read from the tables in the appendix (while *k* affects the variance due to changes in the train-test split size, these effects are well-aligned with the underlying trend).

**Figure 5:**
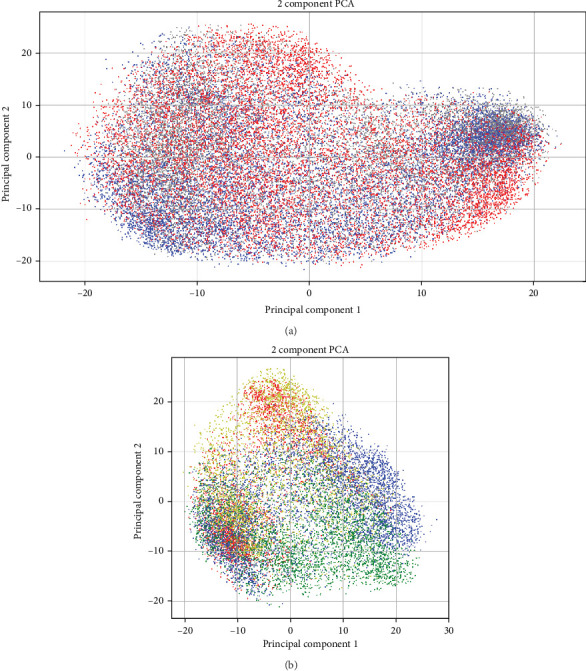
(a) Wav2Vec PCA projection of records of nine female patients divided into three groups based on their depression SUDS score. The groups are indicated by these colors in the plot: red—(SUDS < 2), gray—(2 < = SUDS < = 8), blue—(8 < SUDS). As evident, the generic wav2vec doesn't get any depression signal. (b) PCA projection on two dimensions of Wav2Vec embeddings. Embeddings were extracted per 20 ms segments of audio. Records are of two female patients and two male patients. The colors annotated are red and yellow for female patients and blue and green for male patients. As evident, the signal is dominated by gender and also identity. SUDS, subject unit of distress.

The best accuracy lift over the baseline is very small, at most 2%, which is never more than one std in either case (in many cases, the std is 2%–3%). We suspect the low accuracy lift is because the dominant signal may still be gender and not depression, thus confounding the results. Support for that can be seen in Figure 5a,b. A Wav2Vec embedding of the samples was computed and projected in 2D using PCA. As evident from the figure, the main variance in the data is the gender and identity of the patient, while depression seems to go under the radar of the generic-trained Wav2Vec network (as one would expect).

Wav2Vec is a framework developed by Facebook AI Research for self-supervised learning of speech representations [75]. It leverages raw audio waveforms to learn robust representations by predicting the latent speech units from masked portions of the input. The model has demonstrated impressive performance across various speech recognition benchmarks, advancing the state of the art in automatic speech recognition (ASR). However, as we see in the results, the case of depression is very different.

### 9.1. Results for the LH-Depression Group (Low/High SUDS)

**Figure 6:**
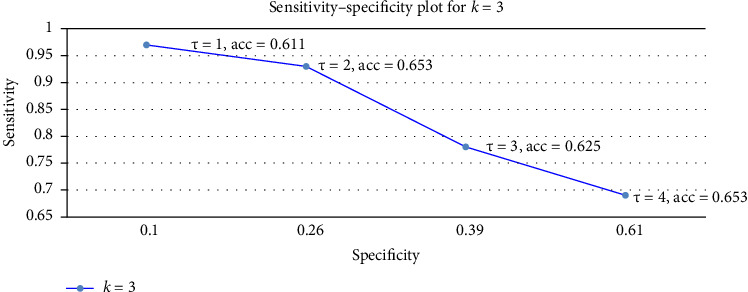
Sensitivity–specificity tradeoff for the LH dataset as the threshold (*τ*) varies from 1 to 4, evaluated using three-fold cross-validation (train set: 34 women, test set: 18). The highest accuracy with the lowest variance is achieved at *τ* = 4 (65.3% ± 2.8%). The baseline accuracy is 50%, with a standard deviation of 6.8%, calculated as the average success rate of a random guess over three tests. Our model outperforms chance by 15.3 percentage points (2.25 standard deviations, *p*-value 0.01). The standard deviation across folds for *τ* = 4 is 2.8%.

We now move to the most promising result, the LH group's performance. The results are shown in Figure 6 for *k* = 3, which we found to be the best tradeoff in size between the test and the train (and not *k* = 4 as in the larger sample, as this leaves a very small 12-women test set). The raw data is given in Table A3 in the appendix.

The best accuracy lift, a 15.3% improvement over the 50% baseline, is obtained at *τ* = 4. This should be compared with the 10% lift reported for DAIC-WOZ, which was achieved with a train set of size 132 [61]. Our lift was achieved in a three-fold cross-validation (3-CV) test, where the train set contained 0.66 × 52 = 34 women and the test set 18 women. To contextualize this improvement, consider an average over three rounds of 18 trials, each with a success probability of 0.5. The expected accuracy in such a setting remains 50%, with a standard deviation given by $\sigma =\frac{1}{18}\sqrt{18\cdot 0.5\cdot 0.5}=0.118$. Averaging over three rounds, the standard deviation of the mean success rate is further scaled down by a factor of $\sqrt{3}$, giving *σ*′ = 0.068, or 6.8%. Our observed 15.3% lift is thus ~2.25*σ*′, or *p*-value of 0.01, emphasizing the statistical significance of our results. The key insight from Table A3 in the appendix is that overall, we get a persistent 10%–19% incremental accuracy improvement over the baseline in all *k*-CV tests and thresholds. The best result is a 19% improvement obtained at a 4-CV test, which makes sense as this is the largest train size (40 women). However, the tradeoff is a higher variance of 5% in the test (12 women). We therefore chose to report the 3-CV test in Figure 3, which balances the train and test set sizes better (36 + 18 women).

### 9.2. Adding SUDs and Pain Scores

We now explore what happens when SUDs are added in addition to the OpenSMILE features.  Table 2 summarizes the results. While SUDs alone give 86% accuracy, adding acoustic features pushes the accuracy to 92.4%. We now proceed with a short statistical analysis that proves that this 6.4% lift is at a *p*-value of 0.1.

Under the null (acoustic features don't add anything on top of SUDs), the expected accuracy is 86%, and the standard deviation of the accuracy for a binomial distribution with *p* = 0.86 and *n* = 26 (we refer to the 2-CV test, with *n* = 26 test size) is

  $$\begin{array}{c} \sigma =\frac{1}{n}\sqrt{n\cdot p\cdot \left(1-p\right)}\approx 0.0685. \end{array}$$

Averaging over two folds further reduces the standard deviation by a factor of $\sqrt{2}$, yielding *σ*′≈0.0484. Our observed lift of 6% corresponds to a *Z*-score of *Z* = (0.92−0.86)/0.0484 ≈ 1.24. The corresponding one-tailed *p*-value is *p* = 1 − Φ (1.24) ≈ 0.1.

Although a *p*-value of 0.1 does not meet the conventional 0.05 threshold, it is still informative in this context. In exploratory research, such values can highlight effects worth further investigation.

### 9.3. Feature Importance Analysis

**Figure 7:**
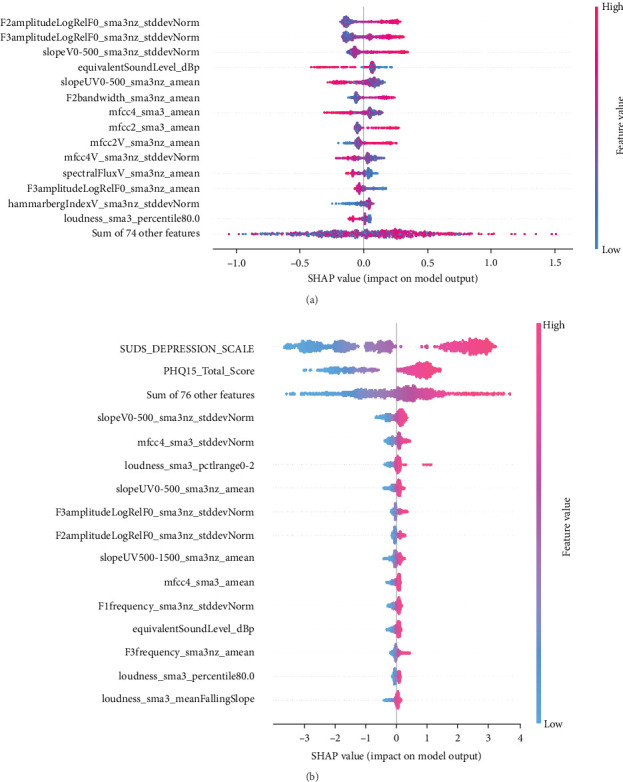
Swarm plot using the SHAP library. Top features are presented. (a) SHAP values when acoustic features are used. (b) SHAP values when acoustic features together with SUDs and PHQ15 (Pain) scores are used. SHAP, SHapley Additive exPlanations; SUDS, subject unit of distress.

Feature importance ranking is crucial to understanding ML models, particularly in identifying which features most significantly influence predictions. SHapley Additive exPlanations (SHAP) is a widely used method for this purpose, providing a unified measure of feature importance based on cooperative game theory [76]. SHAP values explain the contribution of each feature to a model's prediction, making them valuable for interpreting complex models like boosted methods (e.g., our CatBoost pipeline) or deep learning networks. The SHAP summary plot, often visualized as a SHAP swarm plot, succinctly conveys this information. In a SHAP swarm plot, Figure 7, each feature's importance is represented by the spread of SHAP values across all samples, with colors indicating feature values. This visualization highlights the rank of feature importance (the feature names, on the left side of the plot, are ordered by feature importance) and the direction (positive or negative impact) and distribution of each feature's influence, offering a comprehensive view of how features interact within the model. Each row corresponds to a different feature, and all the data is plotted, varying on the horizontal axis. For example, the most important feature in Figure 7a, F2amplitudeLogRelF0_sma3nz_stddevNorm, is on the top row. We see that chunks with higher values of this feature (red colored) have an increased chance of being labeled with depression. The *x*-axis gives the impact on the model's output.

When using a CatBoost model to predict a binary label, the model's output before applying a decision threshold (e.g., 0.5) is in the form of log-odds (also known as the logit) rather than a direct probability. The SHAP values in this context represent the impact on the log-odds of the positive class. To interpret the impact on the probability of the positive class, one can convert the log-odds to a probability using the logistic (sigmoid) function 1/(1 + *e*^logodds^). For instance, if the base log-odds prediction (without the feature's contribution) was 0 (which corresponds to a 50% probability), adding 1.5 to this would result in a log-odds of 1.5, which corresponds to a probability of ~81.9%. A SHAP value of 0.5 means an increase of probability to 62%.

The paper by Janardhan and Kumaresh [77], which discusses several feature selection methods for LLDs of the eGeMAPS feature set, mentions the following features as helpful for distinguishing depressed from non-depressed people: F2amplitudeLogRelF0_sma3nz_stddevNorm, F3amplitudeLogRelF0_sma3nz_stddevNorm, slopeV0-500_sma3nz_stddevNorm, slopeUV0-500_sma3nz_amean. All these features are in our top 5 features in Figure 7a [77].

## 10. Discussion

The association between voice and depression was noted scientifically as early as 1938 [78]. Newman and Mather [78] analyzed the voices of 40 patients and noted differences in speech characteristics between the different groups, both in terms of the selection of words (speech content) and the acoustic characteristics of the voice (prosody). Since then, many papers have studied the capacity of voice signals to reflect depression. We refer readers to several recent reviews or proposals on the topic [31, 68]. These studies have found that acoustic features such as pitch, intensity, and speech rate are influenced by the psychomotor retardation, fatigue, and cognitive impairments typical of depression. For example, some studies on depression detection using voice showed correlations with the fundamental frequency of the complex speech tone—also known as the pitch or F0 [79, 80]. With the advent of ML, correlations were replaced with more powerful predictive methods to show that results generalized well (train-test split) alongside more elaborate performance evaluation metrics (e.g., precision, recall, F1-score).

In this paper, we presented the details of our methodology designed to address the challenges we mentioned when assembling data for a voice-based depression detection pipeline (challenges A–D). We show how meeting these challenges using high-quality voice data for depression detection provides a novel perspective on the trade-offs between dataset size and quality.

Chronic pain and depression are closely linked, creating a complex interplay that significantly impacts an individual's quality of life. Research indicates that chronic pain can lead to the development of depression due to the persistent stress and limitations it imposes on daily activities and overall well-being. This relationship is bidirectional; depression can exacerbate the perception of pain, making it feel more intense and less manageable. According to a study by Bair et al. [81], individuals with chronic pain are significantly more likely to experience depressive symptoms, with estimates suggesting that up to 50% of chronic pain patients suffer from depression. Indeed, the prevalence of depression in our dataset was around 50%, as the literature suggests, thus answering Concern (A) (data imbalance).

In this study, we further explored the integration of acoustic features with SUDs scores to enhance the accuracy of depression detection. We are not aware of previous work that combined SUDs and acoustics, although similar modalities, such as text, were used. Our results reveal that using only acoustic features yields a 15% accuracy lift over the baseline, and employing SUDs alone provides a 36% accuracy lift, reaching 86%. The wide gap between the two feature sets is not surprising, as it is well-known that SUDs are correlated with depression and other mental states [72, 82–84] and that acoustic features provide a considerable lift for datasets of our size. Remarkably, however, when both features are combined, the accuracy increases to 92% for women, reflecting their independent contributions.

To reach clinical-level accuracy, a minimum of 95% is needed. Given that depression prevalence is ~ 5%, this ensures that false positives remain below 5%, the chance-level accuracy threshold. Achieving this requires extensive high-quality data collection, which is time-consuming—especially for carefully curated datasets.

Adding SUD scores to the acoustic features offers an intermediate step in settings where self-reports are valid (i.e., no incentive to misreport), enabling a practical triage tool that combines voice and SUDs. This approach, already tested in our study, allows us to validate the method in real-world settings while continuing to expand our dataset to push accuracy beyond 95%.

## 11. Conclusions

This paper gives an overview of the unique pipeline specifically designed to address several key challenges that have long plagued prosody depression detection research, particularly in voice-based diagnostics. The approach presented here effectively tackles issues related to data imbalance, the quality of diagnostic labels, and the generalizability of models across diverse populations. By analyzing a high-prevalence, high-quality dataset collected from a specialized outpatient clinic setting, we demonstrated that our method can achieve robust depression detection results even with a relatively small sample size.

While our dataset is relatively small, this was a deliberate choice reflecting the high cost of data collection—approximately $50 per sample—due to the use of trained personnel who carried out the collection protocol. The central aim of this work was to demonstrate that a carefully planned and targeted protocol can yield meaningful results even with limited data. Our voice-only model achieved a 15% accuracy lift over chance (*p* = 0.01), underscoring the value of investing in quality over quantity.

Another key highlight of this research is depression detection in non-psychiatric community settings, where individuals may experience significant symptoms but do not seek psychiatric care. We identified such a high-prevalence population, demonstrating that these communities are not just a valuable data source but also a critical use case for rapid, noninvasive, and cost-effective screening tools. This approach enables early detection without requiring a psychiatrist or time-consuming questionnaires, making mental health assessment more accessible and actionable.

Through our findings, we aim to encourage further research and development in the field, ultimately contributing to more effective and accessible mental health diagnostics. This paper describes our research findings in a real-world healthcare clinic using voice as a biomarker for the detection of depression using techniques from ML.

## Figures and Tables

**Table 1:** Data description and summary statistics.

Group	#Samples	% Depressed	Avg recording length in seconds (std)	Avg #Chunks (std)
All	143	62.5	66.73 (17.819)	5.701 (1.969)
Women	120	64.2	67.579 (18.684)	5.805 (2.067)
LH-Women	52	50	68.168 (24.413)	5.827 (2.633)

**Table 2:** LH group with eGeMAPS and SUDs features.

Group	Features	*τ*	k	Accuracy mean (stdev)	Sensitivity mean (stdev)	Specificity mean (stdev)	Baseline
high/low SUDS	eGeMAPS + SUDs	3	2	0.924 (0.050)	0.885 (0.163)	0.958 (0.059)	0.5
high/low SUDS	eGeMAPS + SUDs	3	3	0.896 (0.057)	0.902 (0.092)	0.889 (0.192)	0.5
high/low SUDS	eGeMAPS + SUDs	3	4	0.921 (0.014)	0.889 (0.084)	0.964 (0.071)	0.5

**Table 3:** The results of a k-fold cross-validation test using eGeMAPs features of OpenSMILE.

Group	Features	*τ*	*k*	Accuracy mean (stdev)	Sensitivity mean (stdev)	Specificity mean (stdev)	Baseline
All	eGeMAPS	1	2	0.650 (0.016)	0.923 (0.073)	0.159 (0.096)	0.642
All	eGeMAPS	1	3	0.650 (0.028)	0.924 (0.038)	0.157 (0.153)	0.642
All	eGeMAPS	1	4	0.666 (0.034)	0.949 (0.040)	0.159 (0.087)	0.642
All	eGeMAPS	2	2	0.593 (0.051)	0.772 (0.04)	0.273 (0.064)	0.642
All	eGeMAPS	2	3	0.650 (0.028)	0.809 (0.069)	0.359 (0.198)	0.642
All	eGeMAPS	2	4	0.626 (0.016)	0.823 (0.027)	0.273 (0.074)	0.642
All	eGeMAPS	3	2	0.635 (0.099)	0.773 (0.139)	0.386 (0.032)	0.642
All	eGeMAPS	3	3	0.520 (0.028)	0.607 (0.065)	0.362 (0.149)	0.642
All	eGeMAPS	3	4	0.634 (0.083)	0.747 (0.071)	0.432 (0.136)	0.642
All	eGeMAPS	4	2	0.560 (0.028)	0.556 (0.133)	0.568 (0.161)	0.642
All	eGeMAPS	4	3	0.512 (0.024)	0.546 (0.09)	0.457 (0.099)	0.642
All	eGeMAPS	4	4	0.568 (0.069)	0.568 (0.075)	0.568 (0.087)	0.642

*Note:* All patients, men and women, are used. Different values of the decision threshold are tested, as well as different values of *k*. The results are very close to the baseline, with an accuracy lift of 1.8% > 1std of 1.6% (std computed over the folds) for *k* = 2 and *τ* = 1. Also evident from the table is that as *τ* grows, sensitivity decreases while specificity rises.

**Table 4:** The results of a k-fold cross-validation test using EmoLarge features of OpenSMILE.

Group	Features	*τ*	*k*	Accuracy mean (stdev)	Sensitivity mean (stdev)	Specificity mean (stdev)	Baseline
All	EmoLarge	1	2	0.642 (0.027)	0.924 (0.037)	0.136 (0.054)	0.642
All	EmoLarge	1	3	0.650 (0.014)	0.937 (0.042)	0.138 (0.074)	0.642
All	EmoLarge	1	4	0.658 (0.044)	0.95 (0.071)	0.136 (0.117)	0.642
All	EmoLarge	2	2	0.626 (0.050)	0.847 (0.11)	0.227 (0.064)	0.642
All	EmoLarge	2	3	0.667 (0.014)	0.873 (0.024)	0.295 (0.034)	0.642
All	EmoLarge	2	4	0.593 (0.055)	0.784 (0.113)	0.25 (0.114)	0.642
All	EmoLarge	3	2	0.618 (0.076)	0.722 (0.067)	0.432 (0.096)	0.642
All	EmoLarge	3	3	0.528 (0.11)	0.581 (0.171)	0.428 (0.128)	0.642
All	EmoLarge	3	4	0.593 (0.066)	0.684 (0.187)	0.432 (0.155)	0.642
All	EmoLarge	4	2	0.52 (0.04)	0.57 (0.008)	0.432 (0.096)	0.642
All	EmoLarge	4	3	0.585 (0.024)	0.608 (0.078)	0.546 (0.07)	0.642
All	EmoLarge	4	4	0.528 (0.061)	0.557 (0.031)	0.477 (0.136)	0.642

*Note:* Different values of the decision threshold are tested, as well as different values of *k*. The results are very close to the baseline, with an accuracy lift of 2.5% > 1std of 1.4% (std computed over the folds) for *k* = 2 and *τ* = 3. Also evident from the table is that as *τ* grows, sensitivity decreases while specificity rises.

**Table 5:** LH group with eGeMAPS and EmoLarge features.

Group	Features	*τ*	*k*	Accuracy mean (stdev)	Sensitivity mean (stdev)	Specificity mean (stdev)	Baseline
high/low SUDS	eGeMAPS	1	2	0.61 (0.037)	0.951 (0.033)	0.112 (0.083)	0.5
high/low SUDS	eGeMAPS	1	3	0.611 (0.045)	0.975 (0.050)	0.098 (0.122)	0.5
high/low SUDS	eGeMAPS	1	4	0.604 (0.042)	0.972 (0.056)	0.100 (0.101)	0.5
high/low SUDS	eGeMAPS	2	2	0.625 (0.058)	0.900 (0.044)	0.228 (0.159)	0.5
high/low SUDS	eGeMAPS	2	3	0.653 (0.053)	0.927 (0.095)	0.263 (0.173)	0.5
high/low SUDS	eGeMAPS	2	4	0.688 (0.053)	0.964 (0.074)	0.300 (0.258)	0.5
high/low SUDS	eGeMAPS	3	2	0.625 (0.078)	0.834 (0.070)	0.324 (0.153)	0.5
high/low SUDS	eGeMAPS	3	3	0.625 (0.053)	0.786 (0.091)	0.393 (0.160)	0.5
high/low SUDS	eGeMAPS	3	4	0.646 (0.042)	0.857 (0.110)	0.350 (0.252)	0.5
high/low SUDS	eGeMAPS	4	2	0.615 (0.054)	0.738 (0.072)	0.438 (0.142)	0.5
high/low SUDS	eGeMAPS	4	3	0.653 (0.028)	0.693 (0.077)	0.603 (0.090)	0.5
high/low SUDS	eGeMAPS	4	4	0.646 (0.105)	0.786 (0.082)	0.450 (0.191)	0.5

*Note:* Best accuracy lift is obtained at at *k* = 4 and *τ* = 2. At *k* = 3 and *τ* = 4 slightly lower accuracy is obtained but with much smaller stdev.

## Data Availability

Due to privacy concerns and ethical considerations, the raw data used in this study cannot be made publicly available. However, upon reasonable request and subject to appropriate privacy safeguards, a processed table containing the extracted features can be shared with qualified researchers. Interested parties may contact the corresponding author to initiate this process.
